# Urethral stricture secondary to self-instrumentation due to delusional parasitosis: a case report

**DOI:** 10.1186/s13256-015-0686-5

**Published:** 2015-09-15

**Authors:** Muhammad Fahmi Ismail, Eugene M Cassidy

**Affiliations:** 1 Liaison Psychiatry Department, South Lee Mental Health Service, Cork University Hospital, Wilton Cork, Ireland; 2 Department of Psychiatry, University College Cork, Cork, Ireland

**Keywords:** Delusion of parasitosis, psychosis, delusional parasitosis

## Abstract

**Introduction:**

Delusional parasitosis is a rare psychiatric disorder which often presents with dermatological problems. Delusional parasitosis, which involves urethral self-instrumentation and foreign body insertion, is exceptionally rare. This is the first case report to date that provides a detailed presentation of the urological manifestation of delusional parasitosis with complications associated with repeated self-instrumentation and foreign body insertion, resulting in stricture formation and requiring perineal urethrostomy.

**Case presentation:**

A 45-year-old Irish man was electively admitted for perineal urethrostomy with chronic symptoms of dysuria, haematuria, urethral discharge, and intermittent urinary retention. He reported a 4-year history of intermittent pain, pin-prick biting sensations, and burrowing sensations, and held the belief that his urethra was infested with ticks. He also reported a 2-year history of daily self-instrumentation, mainly injecting an antiseptic using a syringe in an attempt to eliminate the ticks. He was found to have urethral strictures secondary to repeated self-instrumentation. A foreign body was found in his urethra and was removed via cystoscopy. On psychiatric assessment, he displayed a fixed delusion of tick infestation and threatened to surgically remove the tick himself if no intervention was performed. The surgery was postponed due his mental state and he was started on risperidone; he was later transferred to an acute in-patient psychiatric unit. Following a 3-week admission, he reported improvement in his thoughts and distress.

**Conclusions:**

Delusional parasitosis is a rare psychiatric disorder. Self-inflicted urethral foreign bodies in males are rare and have high comorbidity with psychiatric disorders; hence, these patients have a low threshold for referral for psychiatric assessment. The mainstay treatment for delusional parasitosis is second-generation antipsychotic drugs.

## Introduction

Delusional parasitosis is a rare psychiatric disorder which often presents with dermatological problems. Delusional parasitosis involving a body orifice is rare. These patients present with fixed beliefs of infestations despite medical evidence. The prevalence of this disorder is estimated at 80 cases per million, with a yearly incidence of 20 per million [1]. The male-to-female ratio was estimated to be 1:1 in patients younger than 50 years, with female predominance above 50 years [1, 2]. The mean duration of the delusion was found to be 3 years [1].

Delusional parasitosis can be a primary disorder (monohypochondriacal psychosis), or it may be due to a secondary cause such as another psychiatric disorder (schizophrenia, affective or organic psychosis, drug-induced psychosis or intoxication) or a physical illness (vitamin B12 deficiency, pellagra, severe renal disease, diabetes mellitus, multiple sclerosis) [3]. It has also been shown that the majority of patients with delusional parasitosis have a comorbid psychiatric disorder [4]. The majority of patients with delusional parasitosis reported skin infestations with insects, worms, or fibres [5]. Most patients will attempt to treat the infestation themselves and, in some cases, this has resulted in more serious complications [5].

Delusional parasitosis, which involves urethral self-instrumentation and foreign body insertion, is exceptionally rare, especially in males. A retrospective case review conducted by Rahman identified 17 cases of males who self-inflicted urethral foreign bodies over the course of 17 years in the Department of Urology, University of California School of Medicine. Out of the 17 cases, seven were diagnosed with a psychiatric disorder. The most common cause of self-instrumentation was associated with auto-erotic behaviours [6].

This is the first case report to date that provides a detailed presentation of the urological manifestation of delusional parasitosis with complications associated with repeated self-instrumentation and foreign body insertion, resulting in stricture formation and requiring perineal urethrostomy.

## Case presentation

A 45-year-old man was admitted under a urology team for elective cystoscopy and perineal urethrostomy for urethral stricture and intermittent urinary retention. He initially presented to his general practitioner with a 2-year history of dysuria, haematuria, and the passing of blood clots via the urethra. Despite multiple courses of antibiotics and anti-inflammatory agents, they only offered temporary relief and did not resolve his problem. He was referred to the urology team for further investigation. He was electively admitted and had multiple investigations, which included rigid cystoscopy with meatal dilatation, as well as a computed tomography (CT) urethrogram; a urethral biopsy was subsequently performed. He was diagnosed with a stricture affecting the penile urethra.

He was then referred for a second opinion for urethroplasty; however, he was deemed not suitable for the procedure. Since then, he had been experiencing intermittent urinary retention; therefore, a long-term urinary catheter was inserted and it was planned that he would undergo elective perineal urethrostomy to defunction his urethra with the aim of improving his symptoms. The urology team diagnosed that his chronic urological problem was due to repeated self-instrumentation. Upon this admission, our patient had an initial cystoscopy performed, which showed a rigid anterior urethra with a tight circumferential fibrotic penile urethra; a foreign body (a small piece of plastic) was found and removed. There was evidence of necrotic debris secondary to self-instrumentation.

During this admission, our patient told the urology team that he believed there were ticks coming out from his penis and that they had ‘clogged up’ his penis, which caused the pain and retention. The urology team requested a psychiatric evaluation to assess our patient’s mental state and capacity to consent for perineal urethrostomy. On psychiatric assessment, he reported a 4-year history of dysuria, haematuria, urethral discharge, and intermittent urinary retention. On further questioning, he believed that his symptoms were due to tick colonization in his urethra. He was unsure as to how he contracted the parasites, but he believed that the dampness of his house had become a breeding spot for ticks. He reported intermittent penile pain, pin-prick biting sensations in his penis, and a burrowing sensation. He also reported that he has seen the tick from the urethral discharge and that it was as big as few centimetres. He also believed the sediments in his urinary bag were fragments from dead ticks. In addition, he further expressed fear that the ticks would multiply and spread to the rest of his body.

Our patient had been conducting research on the Internet to determine how to fix his problem. To alleviate his symptoms, he had been injecting an antiseptic liquid into his urethra using a syringe three times a day for the last 2 years. He had also has been using a cotton-tipped swab with over-the-counter anti-parasitic ointment, and had inserted them into his urethra to ‘burn’ the ticks out. He also expressed his frustration, and at times he was thinking of ‘cutting out’ and surgically removing the tick. Apart from encapsulated delusional parasitosis and somatic delusions, there was no evidence of any other delusions (paranoid, grandiose, nihilistic, guilt) or hallucinations (visual, auditory, olfactory). There was no evidence to suggest thought interference or passivity phenomenon. He denied any pervasive low mood or any other depressive features. He denied any loss of appetite or weight loss. His function remained unchanged over the last 4 years.

Upon mental state examination, he presented as somewhat dishevelled and maintained intermittent eye contact. He was calm and cooperative throughout the interview. His speech was coherent and spontaneous with a normal rate and volume. There was no latency of response evident. There was evidence of poor cognitive flexibility. His mood was low, but he presented with reactive affect. There was no evidence of a formal thought disorder. There was no observed perplexity or distractibility to suggest overt hallucination. He reported delusional interpretations of his somatic symptoms rather than a true tactile hallucination. He has firm and fixed delusional beliefs that did not respond to challenges. He had poor insight into his condition and his judgement was severely impaired. He was orientated to time, place, and person with preserved attention, recall, and short-term memory. He denied thoughts of self-harm or suicidal ideation. He was expressing thoughts to self-inflict injury; however, he denied any active intent or plan.

He has a history of attending a local community psychiatric service 3 years ago for alcohol abuse and behavioural difficulties. There was no axis one psychiatric diagnosis made at the time, and he only required brief social intervention. He had a previous history of alcohol abuse in the past, but he denies any recent alcohol abuse or drug misuse. He also had minor public disorder offences in the context of alcohol intoxication. He denied any past medical history and he denied any previous diagnosis of sexually transmitted infection. He reported that he stopped schooling at the age of 11 years. He has been unemployed over the last 10 years. He has been living alone throughout his life and does not have any close relationships. He denies any known mental health illness in the family.

Examination under anaesthesia and rigid cystoscopy showed a rigid anterior urethra with a tight circumferential fibrotic penile urethra. Stricturoplasty was performed and a foreign body (a small piece of plastic) was found and removed. A subsequent CT urethrogram was normal and urethral biopsy showed chronic inflammation with a differential diagnosis of balanitis xerotica obliterans (BXO) or self-inflicted trauma. Blood investigations, which included a full blood count, urea and electrolytes, inflammatory markers, and a liver function test, were normal. A brain CT scan was not performed. A thyroid function test and B12, folate, and ferritin levels were normal. The urinary drug screen was negative. Syphilis serology was not performed.

This patient’s presentation was consistent with a non-affective psychosis. He displayed a solitary, encapsulated, monohypochondriacal psychosis with no other features of other psychotic disorder. However, his background history of poor academic attainment and significantly impaired social and occupational function dating back to his early twenties suggest that this is a delusional parasitosis secondary to probable undiagnosed schizophrenia.

### Differential diagnosis

Our differential diagnosis of this patient includes psychotic disorder secondary to schizophrenia, drug-induced psychosis, organic psychosis, mood disorder with psychosis, and schizoid personality disorder.

### Treatment and follow-up

Our patient was commenced on oral risperidone 2mg for 1 day and it was increased to 4mg. He was reluctantly agreeable to taking the antipsychotic medication and he tolerated it well. Following consultation with the urology team, it was decided that the surgery would be postponed until his mental state improved. After 5 days in a surgical ward, he was transferred to an acute in-patient psychiatric ward with a long-term urinary catheter in situ. Our patient was voluntarily admitted to an acute in-patient psychiatric ward. He was continued on oral risperidone 4mg once daily. His mental state improved over a 3-week admission. He indicated that he was less bothered and distressed by his thoughts. He was discharged with a plan for follow-up on an outpatient basis by the community mental health team and urology team.

## Discussion

Delusional parasitosis is a rare psychiatric disorder that presents with fixed beliefs of infestations despite medical evidence. True epidemiological studies in Germany estimated that the prevalence of delusional parasitosis was 80 cases per million with a yearly incidence of 20 per million [1]. Recent epidemiological studies in the United Kingdom reported a point prevalence of 1.5 per million [7]. Psychodermatological presentation remains the most common presentation of delusional parasitosis [7].

Delusional parasitosis can be a primary disorder (monohypochondriacal psychosis), or it may due to a secondary cause such as another psychiatric disorder (schizophrenia, affective or organic psychosis, drug-induced psychosis, or intoxication) or a physical illness (vitamin B12 deficiency, pellagra, severe renal disease, diabetes mellitus, multiple sclerosis) [3].

Delusional parasitosis is more common among those above the age of 50 years and it tends to be insidious in onset [1]. The mean duration of the delusion was found to be 3.0 ± 4.6 years, which is consistent with the findings in this case [1]. One of Trabert’s findings consistent with this case is that social isolation is part of a pre-morbid feature for those with delusional parasitosis [1]. The majority of those who suffer from delusional parasitosis present to dermatologists or other medical specialists [5]. The largest cohort of cases to date thus far was analyzed by Ashley at the Mayo Clinic; of 147 cases of delusional infestation, 98% of cases presented to a dermatologist [5].

The most common area of infestation is primarily the skin. Delusional parasitosis involving body orifices is rare. The most common pathogens that were believed to be the agents of infestations were insects (80%), worms (27%), and fibre (20%) [5]. Some patients with delusional parasitosis may present with ‘evidence’ of infestation in containers, which is known as the specimen sign [8]; which also historically known as the matchbox sign [9]. When the evidence was inspected by the clinician, it was found to mainly consist of skin, skin debris, wounds, and particles from cloth and hair [10]. In this case, our patient insisted that the sediments in his urinary bag were evidence of dead ticks, which is consistent with specimen sign. Most patients will attempt to treat the infestation, and some of these attempts have resulted in serious complications [5].

Self-inflicted urethral foreign bodies in male in urology are rare. A retrospective review of 17 cases identified that seven cases of male self-inflicted urethral foreign bodies in urology were due to psychiatric disorders. Two out of 17 cases had a stricture with a 2-year history of repeated self-instrumentation, and one case required perineal urethrostomy due to progressive retention, which is similar to what was observed in this case. The majority of cases were found to be secondary to auto-erotic behaviours [6]. It is important to emphasise the need to conduct a thorough risk assessment in these patients in order to prevent further damage.

Due to the rarity of self-inflicted urethral instrumentation, a low threshold for psychiatric referral to rule out psychiatric disorders should be adopted. One study reported that patients with delusional parasitosis were highly associated with comorbidities of other psychiatric disorders (up to 74%), and the most common were mood disorders [4]. Due to the high level of comorbidities in these patients, it was recommended that all patients presenting with delusional parasitosis receive psychiatric referral [5].

The cause of delusional parasitosis remains unknown. Dopaminergic overactivity has been proposed primarily in the limbic area, which is similar to schizophrenia. One study looking at the pre- and post-treatment neuroimaging of patients with delusional infestation, while studying the fronto-striato-thalamo-parietal network, showed D_2_ blocking with similar occupancy rates as in schizophrenia [11]. One of the case control studies performed with structural neuroimaging demonstrated abnormal gray and white matter volume, thus supporting a neurobiological model of disrupted prefrontal control over somatosensory representations [12]. Two hypotheses of psychopathogenesis have been proposed. The first theory proposed that this condition involves a tactile hallucination that leads to a secondary delusion, and the second theory proposed that a primary delusion existed and was reinforced by perceptual disturbances [13].

Treatment for a patient with delusional parasitosis is challenging due to a lack of insight and the patient’s reluctance to engage. One survey that was conducted by Lepping in the UK showed that only one-third of patients were prescribed with psychotropic medications [7]. Treatment for delusional parasitosis depends on the pathogenesis. Historically, pimozide has been the antipsychotic of choice, especially in psychodermatological presentations, due to its antihistaminic properties. Pimozide is no longer regarded as a first-line antipsychotic due to concerns associated with its drug safety, and given that similar efficacy can be achieved from second-generation antipsychotics (SGAs) [7].

Despite the current mainstream agreement regarding SGA as first-line treatment for delusional parasitosis, a survey by Lepping in the UK showed that pimozide is by far the most common antipsychotic prescribed by dermatologists [7]. There are no available randomized controlled trials (RCTs) to date, and from a survey of dermatologists in the UK conducted by Lepping, respondents were split with respect to the feasibility of conducting a RCT for the treatment of delusional parasitosis. Many studies have demonstrated the efficacy of SGA - mainly risperidone, olanzapine, and amisulpride - with complete remission achieved in up to 70% of patients [3, 13, 14]. It was also found that secondary delusional parasitosis is more likely than primary delusional parasitosis to respond to SGAs [14]. There was a high association of comorbidities in patients with delusional parasitosis - primarily mood disorders - which may require augmentation with an antidepressant [5].

## Conclusions

Delusional parasitosis is a rare psychiatric disorder. Self-inflicted urethral foreign bodies in males are rare and have high comorbidity with psychiatric disorders; hence, these patients have a low threshold for referral for psychiatric assessment.

## Consent

Written informed consent was obtained from the patient for publication of this case report. A copy of the written consent is available for review by the Editor-in-Chief of this journal.

## Abbreviations

BXO: balanitis xerotica obliterans
CT: computed tomography
RCT: randomized control trials
SGA: second-generation antipsychotic
